# Is Beauty in the Eyes of the Beholder? Aesthetic Quality versus Technical Skill in Movement Evaluation of Tai Chi

**DOI:** 10.1371/journal.pone.0128357

**Published:** 2015-06-05

**Authors:** Paola Zamparo, Elena Zorzi, Sara Marcantoni, Paola Cesari

**Affiliations:** Department of Neurological and Movement Sciences, University of Verona, Verona, Italy; University of Bologna, ITALY

## Abstract

The aim of this study was to compare experts to naïve practitioners in rating the beauty and the technical quality of a Tai Chi sequence observed in video-clips (of high and middle level performances). Our hypothesis are: i) movement evaluation will correlate with the level of skill expressed in the kinematics of the observed action but ii) only experts will be able to unravel the technical component from the aesthetic component of the observed action. The judgments delivered indicate that both expert and non-expert observers are able to discern a good from a mediocre performance; however, as expected, only experts discriminate the technical from the aesthetic component of the action evaluated and do this independently of the level of skill shown by the model (high or middle level performances). Furthermore, the judgments delivered were strongly related to the kinematic variables measured in the observed model, indicating that observers rely on specific movement kinematics (e.g. movement amplitude, jerk and duration) for action evaluation. These results provide evidence of the complementary functional role of visual and motor action representation in movement evaluation and underline the role of expertise in judging the aesthetic quality of movements.

## Introduction

The ability that humans have to “read” the kinematics of an observed action has recently received support and evidence from both behavioural and neuroimaging studies. For movement detection, both motor and visual areas are involved [1] forming together an Action Observation Network (AON), which is believed to work as an internal simulator of the same action when it is observed [2]. Following this idea, several studies indicated that individual experience plays a crucial role in the recognition and simulation of an observed action, and that motor experience in particular can modulate the activation of AON. Indeed, professional dancers are able to decode other dancers’ movements [3] and in sport, the same ability was found to correlate with selective neural activity at the level of the motor cortex [4–7]. From a behavioural point of view, humans have been shown to be highly sensitive to movement and able to understand actions even when presented as simple constellations of moving light points [8], suggesting that an observer relies on specific movement kinematics for action evaluation. When sequences of movements, with known kinematics, were presented to observers who were asked to disentangle good from mediocre dancers, a strong correlation was found between observer evaluations and the following features: movement, speed and amplitude [9], dancing ability and body symmetry [10], positive evaluation of the dance performance and movement amplitude, stability and time (i.e. holding an arabesque for longer) [11].

Interestingly, in sport and dance, a movement can be categorized as having both technical and aesthetic components. It is possible that both components are in principle represented in the kinematics of the movement, and possibly differently recognized and rated by experts or naïf evaluators. Certainly, the technical and aesthetic qualities of human motion are inter-related and conjunctly developed as the skill level increases. This consideration has two main consequences: 1- an improvement in “technical qualities”, which occurs with training and practice, should be associated with a concomitant increase in the “aesthetic qualities” of the movement concerned; and 2- it is difficult to define which movement qualities can be defined as purely technical and which can be considered as purely aesthetic.

However, the distinction between technical and aesthetic movement qualities is “purely operational”, since an aesthetic judgment of human motion seems to be based, at least in part, on some technical aspects of motion. As pointed out by Best [12]: “a smooth flowing style is more highly regarded aesthetically because it appears to require less effort for the same result than a jerky style”. This further suggests that an improvement, through training and practice, of some measurable (e.g. metabolic/biomechanical) qualities should be associated with a concomitant improvement in the aesthetic appreciation of that movement. Indeed, research has shown that through training and practice, the level of muscle co-contractions is reduced [13–15], movement control is improved [14,16–18] and energy expenditure is reduced [19]

However, beauty is also a function of the experience of the perceiver [20]. As shown by Scully [21], both qualified judges and naïve observers, when asked to rate the performance of young female gymnasts, produced similar scores for both aesthetic qualities and level of technical execution. However, while naïve observers equated aesthetic qualities with technical skill, qualified judges differed in the relationship found between these two dimensions. This indicates that expertise affects the perception of relevant information used when judging aesthetic quality.

In the same vein it was shown that athletes are expert observers due to their ability to accurately recognise context-specific movement cues [22]. This knowledge appears fundamental when anticipating actions performed by others [23], when making decisions [24] and when recalling and applying efficient movement strategies [25]. However, little has been done to understand the capacity to disentangle the technical from the aesthetic qualities of a performance, based on the judge’s level of motor skill.

On the basis of all these considerations, we could expect that the ability to evaluate the technical and aesthetic qualities of an observed movement might differ for experts and non-experts. In other words, we would expect that the level of expertise in performing a sequence of movements correlates with the ability to judge the details of the same sequence when observed (e.g. the action details are represented by its movement amplitude, velocity, acceleration and so on). Indeed it has been shown that professional athletes are able to predict the fate of an action due to their capacity to “read the kinematics” of the action observed [4, 8]. To summarise, two main hypothesis are stated: i) movement evaluation will correlate with the level of skills shown in the kinematics of an observed action, such as movement amplitude, jerk and duration but ii) only experts will be able to unravel, independently of the level of performance, the technical from the aesthetic components of an observed action.

## Materials and Methods

The performance under analysis is Tai Chi, a branch of Chinese martial arts. The Yang style investigated in this study is composed of 108 different postures that should be executed with even, slow and steady movements. This study considers the first of the four sequences (named “Lu”) of the Yang style, which is composed of 21 postures and is generally taught in the first year of practice.

Among other factors, the correct execution of these movements requires learning all the postures in the correct way and in the correct sequence; moving from one posture to the next in a continuous and fluid way, and matching the movements to one’s breathing pattern. With years of practice, breathing frequency is reduced, the movements become slower and wider, and the duration of the sequence is increased.

As a result, a Tai Chi performance contains aesthetic and technical qualities that could be identified, the former as fluidity and grace, the latter as the level of technical skill and ability to control balance.

### Participants

Fifty-six participants were recruited and divided into two groups: twenty-six were Tai Chi practitioners (8.2 ± 1.9 years of practice), from now on defined as expert observers (EO) and thirty were non-expert observers (NEO) (see Table 1).

**Table 1:** Main characteristics of the observers (data are means ± SE).

Observers	EO (N = 26)	NEO (N = 30)
Male/Female	14/12	17/13
Age (years)	43.5 ± 2.6	25.0 ± 0.4 *
Experience (years)	8.2 ±1.9	

**Footnote:** Expert observers (EO); non-expert observers (NEO).

* p < 0.05.

The non-expert observers were recruited among the sport science master students at the local University; they had no knowledge of Tai Chi or of martial arts but were all familiar with sports and physical exercises. Expert observers had a higher age compared to non-expert observers (NEO) (*t*_*(54)*_ = 7.33, *p*< 0.001, see Table 1); however, experience, not age, makes a difference in such judgments, as seen in many different instances when the task is action observation/evaluation, especially when the age range is within the “adults category”- see for instance [4, 26–27]. All participants received written and oral instructions before the study and gave their written informed consent to the experimental procedure. The Institutional Review Board (Ethics Committee of the Department of Neurological and Visual Sciences, University of Verona, Italy) approved this research and the investigation was conducted according to the principles expressed in the Declaration of Helsinki.

### Stimuli: Video-clips preparation

The video-clips were prepared by recording the Lu sequence performed by twenty-five Tai Chi performers. All performers were wearing the traditional uniform and their Lu sequence was recorded through a digital camera (DCR-SR35E, Sony, Japan) located at a fixed distance in order to obtain video-clips presenting the same background.

Of the 25 performers, ten can be qualified as “high-level performers” (HLP: 15 ± 1.6 years of Tai Chi practice) and fifteen as “middle-level performers” (MLP: 2.4 ± 0.3 years of Tai Chi practice). Also these participants received written and oral instructions before the study and gave their written informed consent to the experimental procedure.

No significant differences in body mass, stature and age were observed between the high-level and the middle-level performers (independent sample test) whereas both the years of experience (*t*_(23)_ = 9.36, *p*< 0.001) and the hours of training per week (*t*_(23)_ = 3.46, *p* = 0.002) were significantly higher for the high-level performance group (see Table 2).

**Table 2:** Main characteristics of the performers and kinematic components of the video clip performances at “normal speed” (data are means ± SE).

Performers	HLP (N = 10)	MLP (N = 15)
Male/Female	7/3	9/6
Body mass (kg)	68.6 ± 4.6	61.5 ± 3.0
Stature (cm)	172 ± 2.6	169 ± 1.8
Age (years)	42.7 ± 1.6	35.8 ± 3.3
Experience (years)	15.0 ± 1.6	2.4 ± 0.3^¶^
Training (h/week)	6.0 ± 0.5	4.1 ± 0.3^¶^
Time (s)	229 ± 14	175 ± 6.9^¶^
MF (Hz)	0.10 ± 0.006	0.12 ± 0.005^¶^
l_BCOM3D_ (m)	23.9 ± 0.82	21.3 ± 0.74*
j_BCOM3D_ (m ⋅ s^-3^)	2.53 ± 0.13	3.31 ± 0.17^¶^

**Footnote:** HLP: high-level performers; MLP: middle-level performers; MF: Movement Frequency; the 3D path length (l) and jerk (j) of the body centre of mass (BCOM): l_BCOM3D_, j_BCOM3D_.

* p < 0.05.

^¶^ p < 0.001.

The level of performance of these two groups was also determined by means of a kinematic analysis (described in detail in supporting information: S1 Document: Kinematic analysis) which allowed us to calculate the 3D length of the path of motion of the body centre of mass (*l*_*BCOM3D*_, m) and the 3D jerk (*j*_*BCOM3D*_, m ⋅ s^-3^) during the Tai Chi execution. A t-test showed that *l*_*BCOM3D*_ was significantly larger (*t*_(23)_ = 2.35, *p* = 0.029) and *j*_*BCOM3D*_ significantly lower (*t*_(23)_ = -3.52, *p* = 0.002) for the high-level performance group compared to the middle-level performance group (see Table 2). Moreover, the time of execution (*TE*, s) of the Lu sequence was significantly longer (*t*_(23)_ = 3.61, *p* = 0.002) and the movement frequency (*MF*, Hz: the number of postures to *TE* ratio) significantly lower (*t*_(23)_ = -3.72, *p* = 0.001) for the high-level performance group compared to the middle-level performance group (see Table 2). Thus, as expected, with the years of practice, the movements become smoother and wider and the movement frequency decreases while the time of execution increases.

For the observational task, to avoid attention loss, only a selected portion of each video-clip was used (from the 10^th^ to 20^th^ posture) and each video-clip was temporally aligned to the others presenting an average duration of 63.6 ± 10.7 s. The video-clips were cut by means of appropriate software (AVS Video Editor 4) and then converted into a Windows Media Video format.

### Procedure

All observers were asked to watch each video-clip and evaluate the performance by means of a Visual Analogic Scale (VAS)[28] ranging from MIN = 0: very poor execution to MAX = 10: very good execution. More specifically, to evaluate the technical qualities of the action observed, the participants were asked to fill in two VAS responses: one evaluating the level of balance control (BC) and one the level of technical skill (TS). In the same way, to judge the aesthetic qualities of the action observed, the participants were asked to fill in two other VAS responses: one evaluating the level of grace and beauty (G/B) and one to evaluate the level of fluidity and continuity (F/C).

Before data collection, all observers underwent a brief introduction about the use of the VAS scale and were shown two video-clips, one of an excellent Tai Chi performer (20 years of practice) and one of a poor performer (1 year of practice). Thus, as suggested by Scully [21], the judgement of the observers was based on “a priori presentation of a standard”. During the observation of these two video-clips no feedback was given, the only aim being to indicate the two extremes of the range of values available (from MIN = 0 to MAX = 10) on the VAS scale. The presentation order of the video clips was randomized across participants. As indicated above, the time of execution of the Lu sequence was significantly longer and the movement frequency lower in more skilled subjects (in HLP compared to MLP). To check whether velocity in action execution affects movement evaluation, we manipulated the speed of the video-clips. The same video-clips were both speeded up and slowed down by modifying their frame rate by means of appropriate software (Virtual Dub 1.9.4). We thus obtained two additional sets of video-clips, one at a frame rate of 50 Hz (“fast speed”) and one at a frame rate of 12.5 Hz (“slow speed”); the original videos (“normal speed”) were recorded at 25 Hz). Only non-expert observers were asked to judge the modified video-clips (with the aid of the VAS scale).

Video clips at normal, fast and slow speed of a high level performance are reported as supporting information; a PLOS consent form was signed by this participant.

### Data Analysis

A 2X2X2 ANOVA with repeated measurements was applied in order to analyse the VAS scale data, considering the 2 levels for Performance (high and middle) and the 2 levels for Movement Qualities (technical and aesthetic) as within-subject factors, and as a between-subject factor the 2 groups of Observers (experts and non experts).

For the data obtained from the non-expert observers in judging the performance at the three different video-clip speeds, an ANOVA with repeated measurements was applied considering the 3 Video Velocities (normal, slow, fast) and for a between-subjects factor the 2 levels of Performance (high and middle). Post-hoc comparisons were performed by means of t-tests applying the Bonferroni correction for multiple comparisons when required.

Linear regressions were computed by the method of least squares to investigate the relationship between VAS scale data and kinematic variables; the correlation coefficient (*R*) was used to indicate the goodness of fit. In all analyses, the significance level was set at *p*< 0.050. Data are reported as means ± SE.

## Results

### Video-clips at “normal speed”

The descriptive statistics of the observed variables is reported in Table 3. We considered as *technical quality* the combined VAS evaluations of the technical skill level and control of balance and as *aesthetic quality* the combined VAS evaluations of the movement grace/beauty and fluidity/continuity. The statistical design (ANOVA with Repeated Measurements) considered the level of Performance (high and middle) and Movement Qualities (technical and aesthetical) as within factors, and group of Observers (experts and non experts) as a between factor.

**Table 3:** VAS scale (0–10) descriptive statistics (data are means ± SE).

		Technical quality		Aesthetic quality	
		TS	BC	G/B	F/G
EO	HLP	5.03 ± 1.34	5.28 ± 1.35	4.89 ± 1.40	4.82 ± 1.53
	MLP	3.21 ± 1.12	3.37 ± 1.20	3.16 ± 1.21	2.92 ± 1.25
NEO	HLP	6.37 ± 0.45	6.40 ±0.52	6.31 ± 0.34	6.39 ± 0.47
	MLP	3.99 ± 0.66	4.04 ± 0.48	3.94 ± 0.47	3.88 ± 0.44

**Footnote:** EO: expert observers; NEO: non-expert observers; HLP: high-level performance; MLP: middle-level performance; TS: technical skill; BC: balance control; G/B: grace/beauty; F/C: fluidity/continuity.

**Fig 1:**
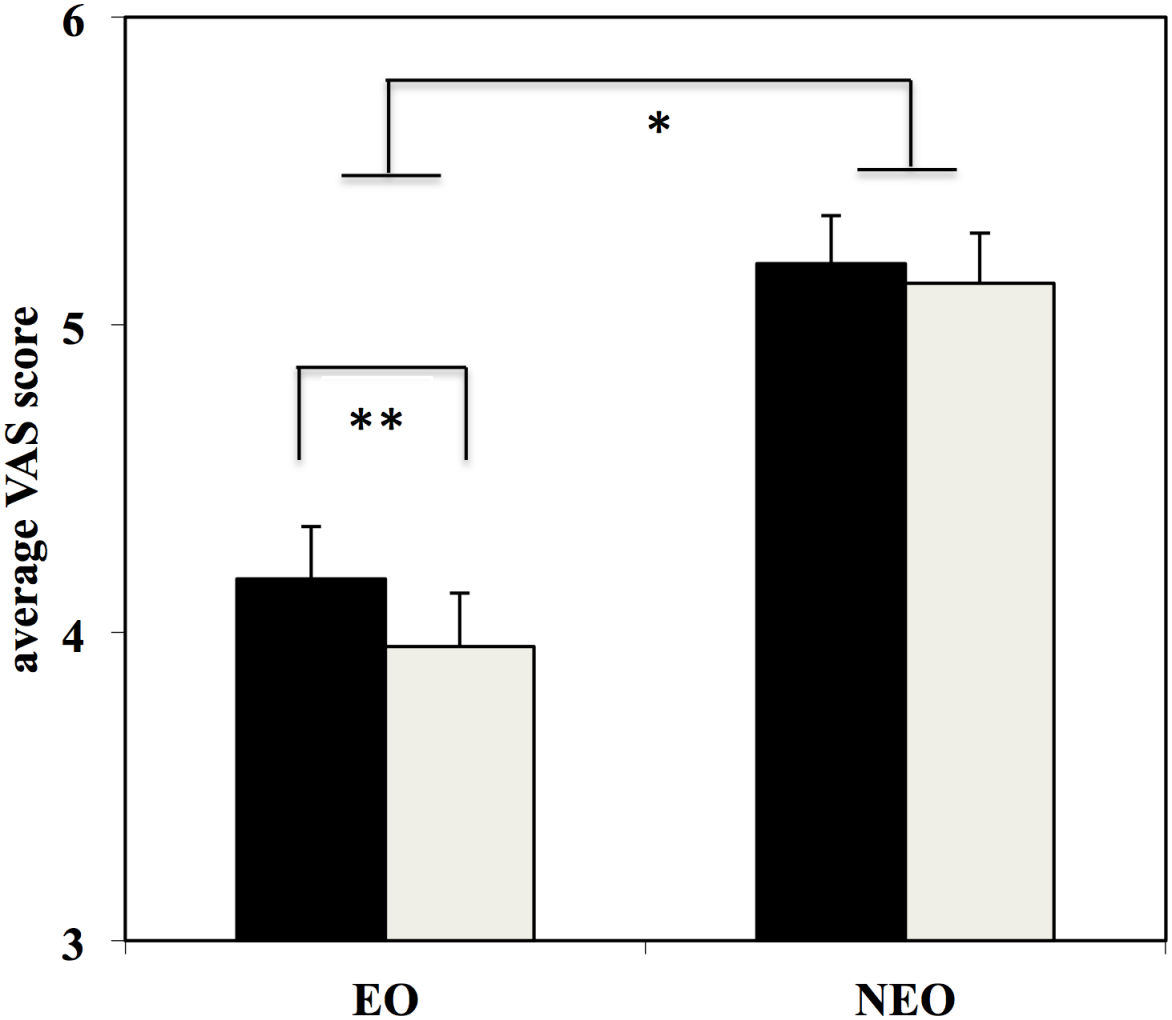
Average VAS score for the two groups of observers (expert observers: EO; non expert observers: NEO). Black columns represent evaluations for technical movement qualities while white columns indicate evaluations for aesthetic movement qualities. Bars represent 1 SE. * indicates significant differences between groups (EO and NEO); ** indicates significant differences between aesthetic and technical qualities.

A main effect for group was significant. Expert observers gave lower scores than non experts observers (*F*_(1, 54)_ = 22.81, *p*< 0.001, \eta_*P*_^*2*^ = 0.297). For the within-subjects analysis a significant effect was found for the level of Performance (high and middle): *F*_(1, 54)_ = 511.70, *p*< 0.001, \eta_*P*_^*2*^ = 0.905 and for the 2 Movement Qualities: (technical and aesthetic): *F*_(1, 162)_ = 18.39, *p*< 0.001, \eta_*P*_^*2*^ = 0.254. The high-level performance received a higher evaluation compared to the middle-level performance and the two qualities of the movement observed were evaluated differently. Importantly, the interaction between the 2 levels of Performance x the 2 Groups of Observers, and the interaction between the 2 Movement Qualities x the 2 Groups of Observers were significant (respectively *F*_(1, 54)_ = 8.94, *p*< 0.010, \eta_*P*_^*2*^ = 0.142 and *F*_(1, 54)_ = 6.52, *p*< 0.050, \eta_*P*_^*2*^ = 0.108). The post hoc comparisons for the former interaction indicated that the level of the Performance was clearly distinguished by both groups (*p*< 0.001) (see Table 3 and Fig 1). The latter interaction showed that while expert observers gave a different evaluation to Movement Qualities (*p*< 0.001), non-expert observers evaluated the two qualities in a similar way (*p* = 0.208) (see Table 3 and Fig 1). Interestingly, the 3-way interaction was not significant (*p* = 0.301), indicating that expert observers were using the same type of evaluation between the two Movement Qualities irrespective of whether they were observing a high-level or middle-level performance.

**Fig 2:**
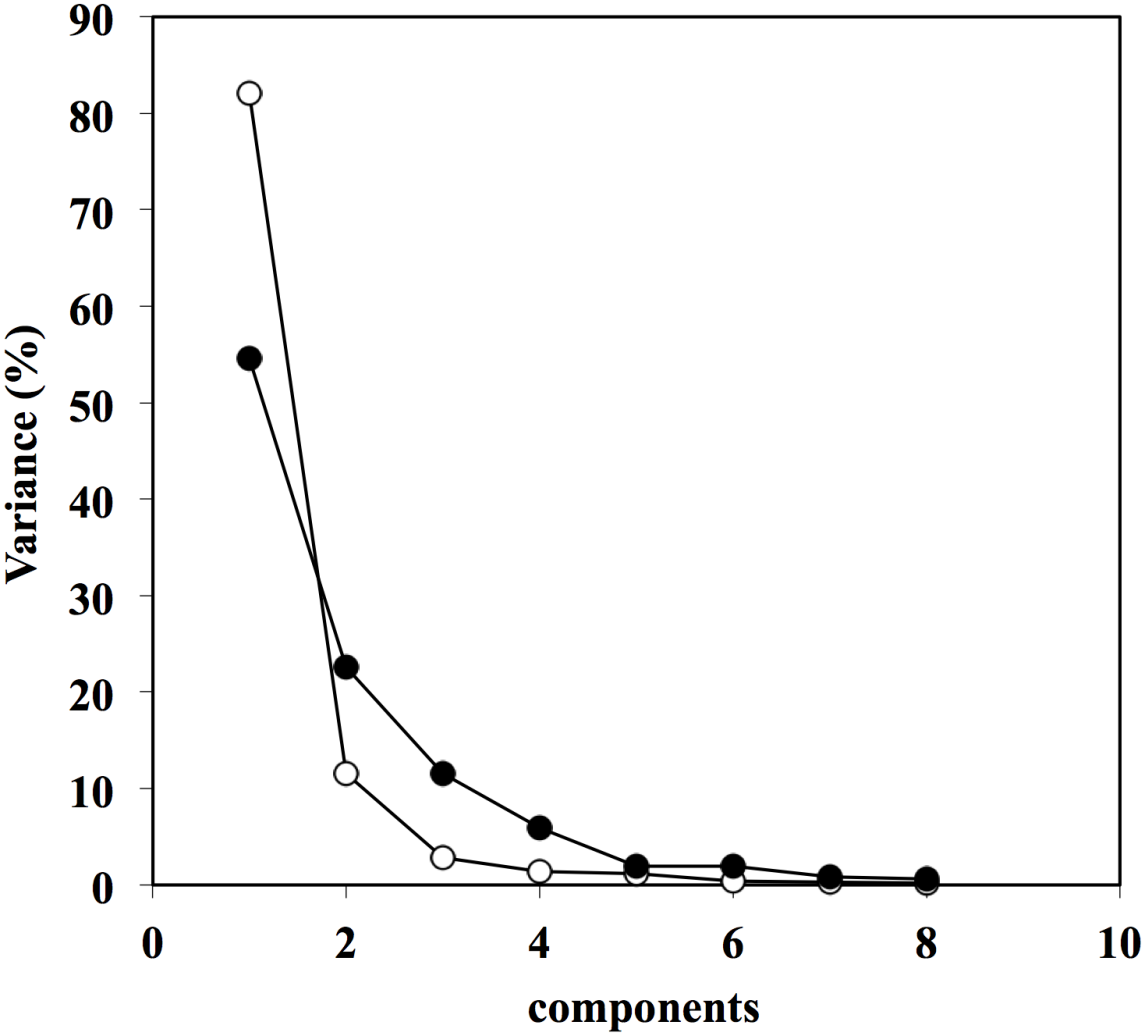
Principal component analysis (PCA). The graph represents the number of PCA components considered and the related percentage of variance. Notice the first component explains 85% of the variance for expert observers (EO) but only 56% of the variance for non expert observers (NEO).

A principal Component Analysis (PCA) was then performed to test the data’s variability components. Principal component analysis (PCA) is a mathematical procedure that transforms a number of possibly correlated variables into a smaller number of uncorrelated variables called principal components. The first principal component accounts for as much of the variability in the data as possible, and each succeeding component accounts for as much of the remaining variability as possible. We applied PCA, considering each group of observers separately, and the analysis (see Fig 2) showed that while for the group of expert observers the first component was able to explain more than 82% of the variance (followed by the second that in total accounted for more than 93% of the variability of the data), for the non expert group of observers the first component was able to explain only 56% of the variance (with an additional 23% by adding the second component with a total of 77%). The number of components needed to capture the total variance, is an index of regularity and stability in the data. In this case, expert observers showed a high level of “coherence” in the judgments delivered when compared to non-expert observers. If two components are necessary to explain 93% of the variability accounted for the experts group, four components are necessary to reach the same amount for the group of non-experts.

### Correlation between the VAS score and movement kinematics

**Fig 3:**
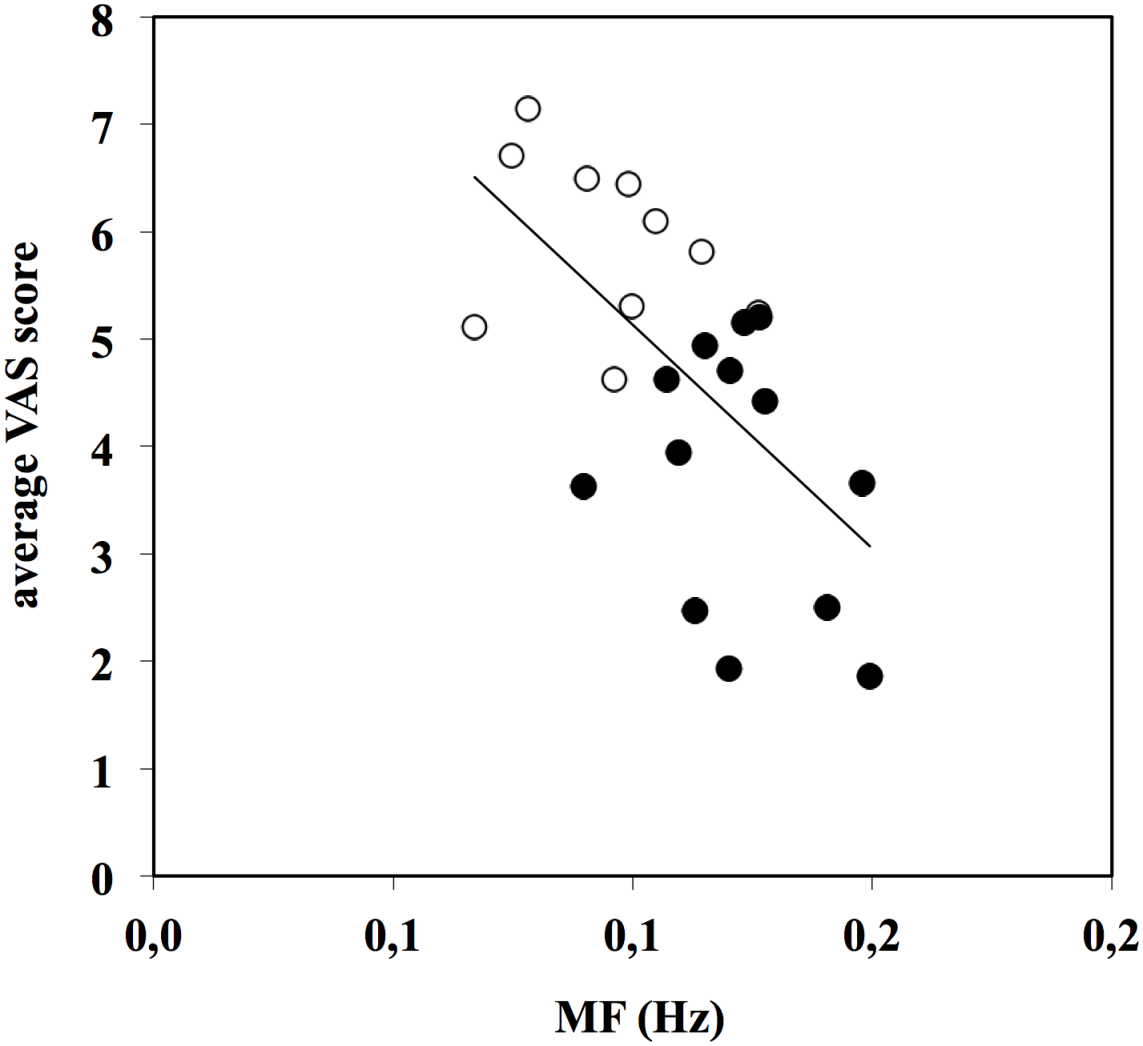
Relationship between VAS score and movement frequency (MF). VAS score = 9.29–41.6 ⋅ MF (*R* = 0.609, *N* = 25, *p*< 0.001). Data points are the grand averages of the scores given by both expert and non-expert observers for the four investigated movement characteristics (open dots: high-level performance, full dots: middle-level performance).

**Fig 4:**
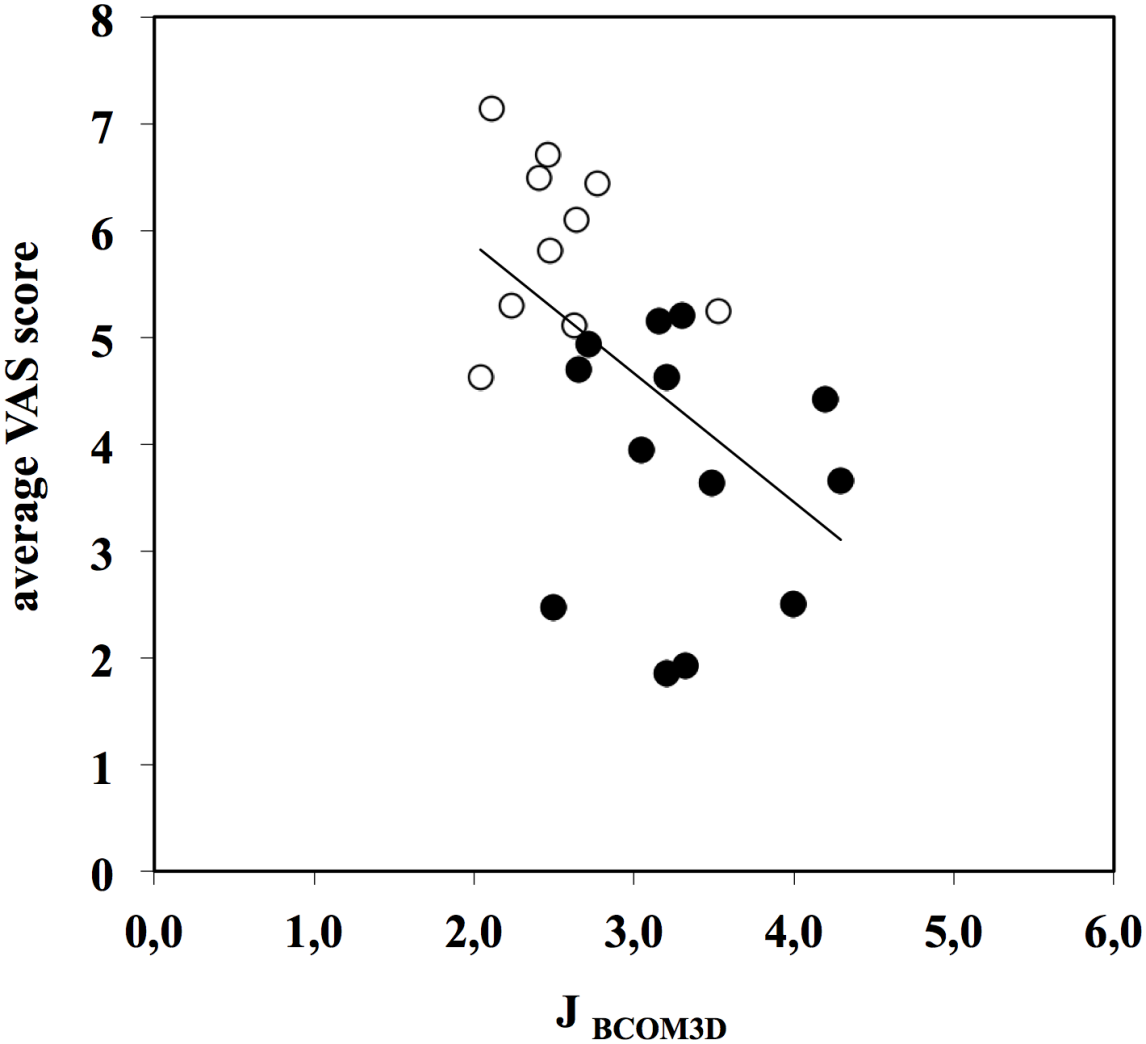
Relationship between VAS score and jerk of BCOM (j_BCOM3D_). VAS score = 8.28–1.21 ⋅ j_BCOM3D_ (*R* = 0.511, *N* = 25, *p*< 0.05). Data points are the grand averages of the scores given by both expert and non-expert observers for the four investigated movement characteristics (open dots: high-level performance, full dots: middle-level performance).

Linear regressions were analysed to check for correlations between the judgments delivered by the observers by means of the VAS score, and the kinematic variables analysed in the models. Significant relationships were found between the VAS score (the combined VAS evaluations of the two technical and the two aesthetic movement characteristics) delivered by the observers and the time of execution (*VAS* = 0.944 + 0.019 ⋅ *TE*; *R* = 0.579, *N* = 25, *p*< 0.001), the movement frequency (*VAS* = 9.29–41.6 ⋅ *MF*; *R* = 0.609, *N* = 25, *p*< 0.001), the length of BCOM path (*VAS* = -2.25 + 0.31 ⋅ *l*_*BCOM3D*_; *R* = 0.664, *N* = 25, *p*< 0.001) and the BCOM jerk (*VAS* = 8.28–1.21 ⋅ *j*_*BCOM3D*_, *R* = 0.511, *N* = 25, *p*< 0.05). These relationships show that jerky movements and high frequency movements are evaluated as less pleasant, while slow and wide movements are considered more pleasant. The relationship between VAS score and movement frequency is reported in Fig 3; the relationship between VAS score and BCOM jerk in Fig 4.

### Video-clips presented at “modified speed”

High-level performances are characterized by a longer time of execution and by a lower movement frequency. If time and frequency are relevant variables that guide the sense of beauty in action evaluation, then by slowing down the video-clips, performance should be rated as more pleasant. We asked non-expert observers to evaluate the same video-clips but this time presented at different speeds (12.5 Hz and 50 Hz). Analysis showed that there was no significant main effect for video velocity (*p*> 0.050) but a significant main effect for level of performance (high and middle) (*F*_(1, 23)_ = 24.12, *p*< 0.001, \eta_*P*_^*2*^ = 0.512) and a significant interaction for video velocity x performance (*F*_(2, 46)_ = 3.27, *p*< 0.050, \eta_*P*_^*2*^ = 0.125).

**Fig 5:**
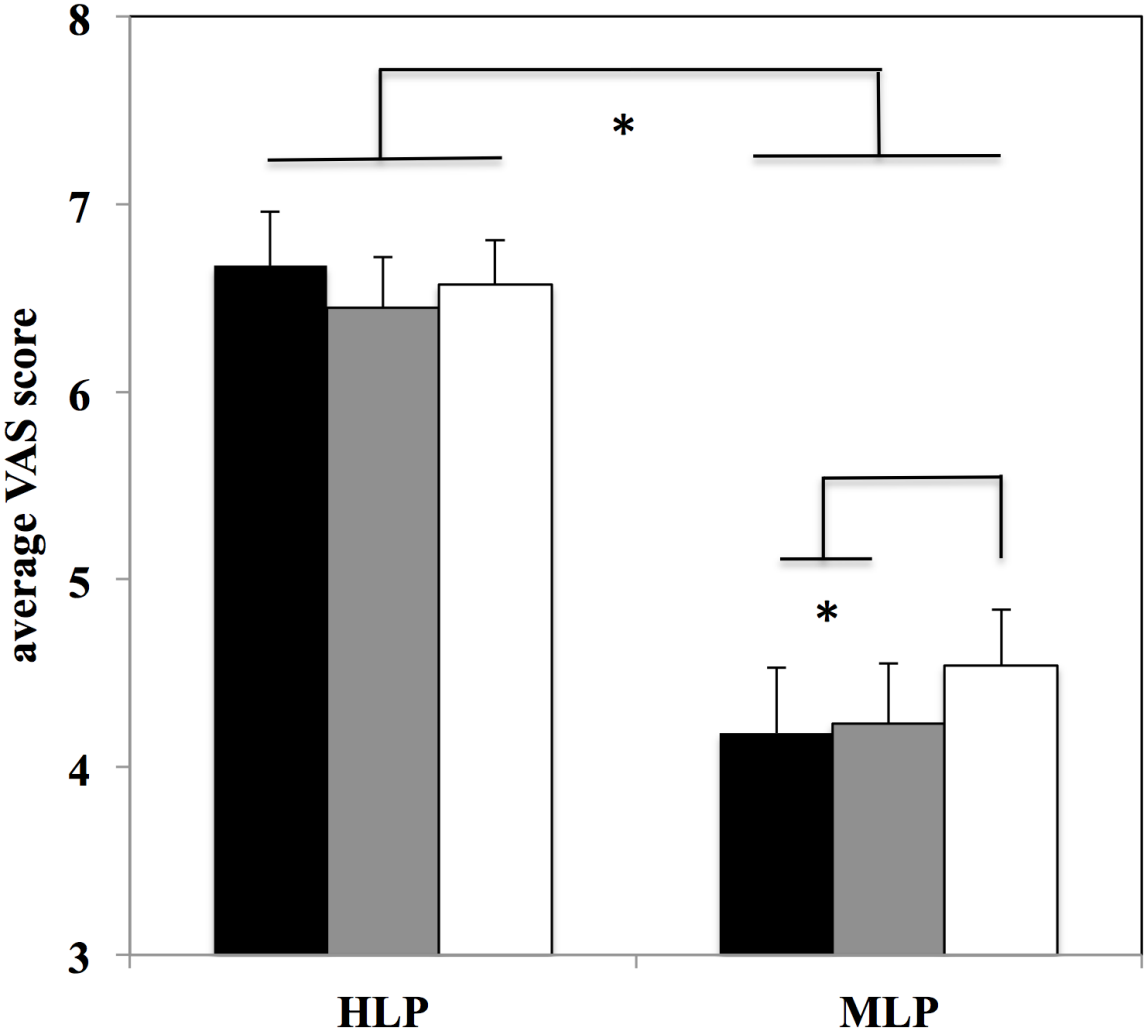
Average VAS scores given by non-expert observers (NEO) to high-level (HLP) and middle-level performances (MLP) at different video speeds. White columns: slow speed (12.5 Hz); grey columns: fast speed (50 Hz); black columns: normal speed (25 Hz). Bars represent 1 SE. * indicates significant differences between video-clips velocities and between levels of performance.

The post hoc analysis indicated that (non expert) observers gave the same evaluation to the high-level performance group independently of the speed of the video presentations, while they differentiated their evaluations for the middle-level performance group. More specifically, pair-wise comparisons showed that only when evaluating middle-level performances, judgments were similar for the sequences observed at normal and at fast speed (*p*> 0.050). On the contrary, judgments became higher when the performances were presented at a slow speed (compared with normal *p* = 0.048 and with fast *p* = 0.001) (see Fig 5). Time of execution (and movement frequency) would indeed seem to be relevant and readily visible variables picked up by the non-expert observers to discriminate between levels of execution.

## Discussion

In this study we address the question of whether the aesthetic and technical evaluation of an observed action could be attributed to specific characteristics of the movement and whether it depends on the level of skill that the evaluator has concerning that movement. More specifically we asked whether aesthetic and technical judgements concerning a human performance are interrelated and depend on the level of action skill possessed by the evaluator when judging the same action.

Tai Chi is not properly speaking an “aesthetic sport” since there is no purpose in being graceful. Nevertheless, it incorporates some features that could be perceived as aesthetically pleasing. Correct Tai Chi execution requires moving from one posture to another in a continuous and fluid way and coordinating postural changes with breathing frequency. With practice, the latter is expected to decrease, the movements to slow down and the time in performing the entire sequence to increase. Thus, this seems an optimal “condition” to test the validity of our hypotheses.

Data reported in this study indicate that: 1) aesthetic qualities judged in terms of continuity/fluidity and beauty/grace were significantly related to the technical qualities judged as technical skill and balance control for both groups of observers; 2) there are indeed features of human movements (at least in Tai Chi) that are recognised as beautiful such as fluency and rhythm (i.e. movement frequency); and 3) expert and non-expert observers showed distinct judgements about the movements observed. As far as point 1 is concerned, it has been shown that both body movement symmetry and a smooth continuation of movements are crucial for a higher aesthetic rating [26]. For point 2, no significant differences were observed in scoring beauty/grace and continuity/fluidity for both groups of observers. This indicates that these characteristics are perceived in a similar way. As far as point 3 is concerned, in general, expert observers presented a tendency to apply lower scores than non-expert observers but, more importantly, expert observers were able to differentiate their evaluations by giving a higher score to the technical qualities (in particular to the movement characteristic called ‘balance control’) and a lower score to the aesthetic ones. On the contrary, non-expert observers evaluated the two movement qualities in a similar way.

The fact that non-experts applied higher scores than experts reflects the well-known propensity to over-evaluate performances considered difficult to execute [2]. However, the main point is that with only a few years of practice, a Tai Chi practitioner is able to distinguish between qualities of movement while a non-expert observer does not have any knowledge of how the sequence has to be performed, and as a result he/she simply equates the aesthetic and technical scores. Similar results were obtained by Scully [21], although she investigated the gymnastic beam routine, which is a sport where the “aesthetic” components are normally included in the overall evaluation, with the result that a tight relationship between aesthetics and level of skill would be largely expected. Data reported in this study confirm her conclusions and extend them to other forms of human movement (e. g. Tai Chi), for which an aesthetic value is not specifically sought.

Our results are sustained by recent works in cognitive neuroscience showing that understanding an action lies in decoding biological motion, by “reading” and internally simulating the kinematics of the action observed [4, 29]. Interestingly, Aglioti and colleagues [4] found that observational ability was higher for basketball players compared to basketball journalists when understanding and anticipating a basketball throw observed on a video-clip. The fact that only Tai Chi experts were able to disentangle the different qualities of movement supports the notion that motor experience develops the ability to recognise and then recall relevant information extracted by observing structured sequences of actions [25, 26,30]. These abilities have been shown to be necessary for decision-making [24] and allow the understanding of high-level information about movement temporal relationships [31].

Likewise here we showed that physical experience can improve perceptual ability in action evaluation and that only practitioners (expert observers in this study) were able to discern between the different components of movements in their evaluations.

Finally, our data suggests that while rating a Tai Chi performance, movement velocity seems to have some importance: high-level performances were characterized as slow-moving. To further test this hypothesis we asked our observers to judge the video clips at different speeds. The results support the idea that beauty is strongly related to kinematic variables measured in the model observed (such as smoothness and frequency). We showed that only when the video-clips were artificially slowed down (but not when speeded up) the observers gave a higher score in their evaluations and only for the middle-level performance and not the high-level one.

Finally we would like to underline the fact that, even if sequences of movements were utilized also in other studies (e. g. to investigate the implicit learning of structured dance movements [32]), to the best of our knowledge this is the first study that has used choreography presented in video-clips to evaluate an individual’s sense of beauty whereas experiments on aesthetics have largely used static visual stimuli or apparent biological motion stimuli produced by static sequences of visual stimuli [26].

## Conclusions

On the basis of the results reported in this study, we can therefore suggest that: 1) among several movement characteristics that may influence the judgment of an observed action, we can list: technical skill, body balance and fluidity/continuity; ii) the level of motor experience possessed by the observer does affect the judgment delivered since; iii) expert and non-expert observers seem to use different strategies when evaluating the aesthetic and technical qualities of movement. Future analysis of pure purposive sports are necessary to investigate whether the equation technical skill = aesthetic quality could be considered a general rule for human movement evaluation.

## Supporting Information

S1 Document Kinematic analysis. (DOCX)

S1 Video A Tai Chi performance at normal speed (25 Hz). (WMV)

S2 Video A Tai Chi performance at slow speed (12.5 Hz). (WMV)

S3 Video A Tai Chi performance at fast speed (50 Hz). (WMV)
