# Supplementary material for: Is Beauty in the Eyes of the Beholder? Aesthetic Quality versus Technical Skill in Movement Evaluation of Tai Chi
Source: PLoS One. 2015 Jun 5;10(6):e0128357. doi: 10.1371/journal.pone.0128357 (PMC4457604; doi:10.1371/journal.pone.0128357)
Supplement: S1 Document — (DOCX) [file pone.0128357.s001.docx]

**S2. Kinematic analysis**

Kinematic data were collected by means of an optoelectronic motion capture system (MX Ultranet, VICON, USA) with a sampling frequency of 100 Hz. The calibrated volume was 4×2×2 m and the disposition of the eight infrared emitting cameras (MX 13, VICON, USA) allowed to detect the position of retro-reflective markers (14 mm in diameter) placed on the left and right side of the participants on specific anatomical landmarks: Cheekbone (HD), Fifth Lumbar Vertebra (L5), Posterior and Anterior Iliac Spines (PSIS, ASIS), Seventh Cervical Vertebra (C7), Acromion Bone (A), Lateral and Medial Homer Condyles (LC, LM), Radial styloid process (SR), Ulnar styloid process (SU), First and Fifth metacarpal (HI, HV), Greater Trochanter (GT), Lateral and Medial Femoral Epicondyles (LE, ME), Lateral and Medial Tibia Condyles (HF, HP), Lateral and Medial Malleoli (LM, MM), Trochlear Calcaneus Process (CA), First and Fifth Metatarsal Heads (IMH, VMH).

Data analysis was performed using the Matlab 7.1 software (Mathworks Inc., Natick, Massachusetts, USA). Kinematic data were interpolated and digitally low-pass filtered at 5 Hz using a fourth-order Butterworth filter. All trials were cut at specific frames: the start corresponded to the “Opening form of Tai Chi” and the end to the “Return to normal” position. The duration of the cutted trials is indicated in the paper as the time of execution (*TE*, s).

From total body kinematics the position of the body center of mass (BCOM) was calculated; the mass of each body segment was estimated according to Dempster et al. [1]; the position of the center of mass of each segment was calculated according to Drillis and Contini [2]; the changes in position of BCOM as well as its velocity, acceleration and jerk were then computed. More specifically, from the matrix of BCOM displacements we obtained the matrixes of velocities, accelerations and jerks, through the differentiation process (frame by frame), each row representing the vector associated to each frame, The norm for each row was then calculated to obtain the intensity of *v*, *a* and *j* for each frame. The average intensity of this matrix of norm vectors was finally calculated.

Based on these data, the 3D path length of the body center of mass (*l _BCOM3D_*, m) was calculated by means of customized Matlab program. The average 3D velocity (*v*, m ^.^ s^-1^), acceleration (*a*, m ^.^ s^-2^) and jerk (*j*, m ^.^ s^-3^) of BCOM were then calculated by knowing the duration of the exercise (*TE*, s). In this paper only data of *l _BCOM3D_* and *j _BCOM3D_* are reported.

**References**

1. Dempster WT, Gabel WC, Felts WJL. The anthropometry of manual work space for the seated subject. Am J Phys Anthropol. 1959; 17: 289-317.
2. Drillis R, Contini R. Body Segment Parameters. Office of Vocational Rehabilitation, Department of Health, Education and Welfare, Report 1166-03. N.Y. University School of Engineering and Science, New York, NY; 1996.
